# Effect of Herbal Therapy to Intensity Chemotherapy-Induced Nausea and Vomiting in Cancer Patients.

**DOI:** 10.5812/ircmj.4392

**Published:** 2013-02-05

**Authors:** Akram Sadat Montazeri, Mehdi Raei, Atefeh Ghanbari, Ali Dadgari, Azam Sadat Montazeri, Azam Hamidzadeh

**Affiliations:** 1 School of Nursing and Midwifery, Shahroud University of Medical Sciences, Shahroud, IR Iran; 2 Clinical Research Development Center, Qom University of Medical Sciences, Qom, IR Iran; 3 School of Nursing and Midwifery, Guilan University of Medical Sciences, Rasht, IR Iran; 4 School of Pharmacy, Tehran University of Medical Sciences, Tehran, IR Iran

**Keywords:** Cancer, Nausea, Vomiting, Herbal Therapy, Ginger, Chemotherapy

## Abstract

**Background:**

Chemotherapy-induced nausea and vomiting are the most important complications for cancer patients as its prevalence has been reported to be about 54-96 percent. ginger has been used for medicinal purposes including nausea and vomiting in traditional Persian, Chinese and Indian pharmacopoeia.

**Objectives:**

The objective of this study was to evaluate the efficacy of complimentary ginger among cancer patients experiencing nausea and vomiting.

**Material and Methods:**

A randomized cross-over clinical trial was carried out on patients under chemotherapy treatment for at least 2 episodes of chemotherapy and at least 2 episodes of previous experience of nausea and vomiting. Subjects of this study received 2 different complementary regimes with 250mg ginger capsule in regime A and placebo capsule in regime B. subjects of the study were crossed over to receive the other regime during the two cycles of chemotherapy.

**Results:**

Findings of the study indicated that subjects receiving ginger showed significant reduction in frequency and intensity of nausea and vomiting compared to placebo receiving subjects.

**Conclusions:**

According to finding of this study, in accordance to most of other researches, ginger is an effective agent to reduce chemotherapy-induced nausea and vomiting. However, there are some researches supporting ginger as a moderate antiemetic agent among cancerous patients under chemotherapy.

## 1. Background

Chemotherapy - induced nausea and vomiting are the most common intensive side effects and an important source of anxiety among cancer patients as its prevalence has been reported about 54 - 96 percent (1). Chemotherapy - induced nausea, retching, and vomiting (CINV) has historically had significant negative impacts on the quality of life (QoL) and daily functioning of patients receiving chemotherapy (2). Though enumeration of vomiting episodes and ratings of nausea are useful for evaluating the clinical efficacy of antiemetics, these measures do not assess the full impact of CINV on the daily life of patients (e.g., daily functioning, appetite, family life, etc.) and thus are not capable of demonstrating the broader impact of treatment. The factors most important to patients may have direct impact on their future health-care decisions and willingness to continue treatment. Patients consistently rank nausea and vomiting as one of the most distressing side effects of cancer chemotherapy (3). Direct expenditures consist of long hospitalization and extra expenditures including medical and nursing care. Whereas indirect costs consist of loosing or decreasing of income dependent on patients, their family members, or people who take care of them. Recently, lot of different methods, pharmacotherapy and complimentary therapies, have been applied for controlling nausea and vomiting. Planning and prescription of suitable therapy, Pharmacological or non - Pharmacological, will considerably improve the quality of life and functional of patients and will positively affect their lives (4). Despite the widespread use of the 5-HT3 receptor antagonist antiemetics, ondansetron (Zofran®), granistron (Kytril®), and dolasetron mesylate (Anzemet®), post chemotherapy nausea and vomiting continue to be reported by over 70% of patients (5).

Since, the substitute and complementary therapies may be used as therapy or supportive methods. These methods can be applied as independent therapy methods or along with other standard methods for cancer patients (3). Historically, plants have been and are still being used for a variety of purposes in all human cultures; food, culinary spices, medicinal, cosmetics, spiritual and ornamental. In the 1990s, WHO has estimated that an impressive 80% of the world population in developing countries relies mainly on traditional practitioners and medicinal plants to meet their primary health care needs. According to a systematic review conducted by Ernst and Cassileth (1998), the average prevalence of CAM therapies across adult patients with cancer from 26 surveys was 31.4%. More recent studies have reported an even higher prevalence of between 70 and 80%. Concurrent use of herbs, herbal products and nutraceuticals may mimic, magnify or oppose the effect of drugs (6). Ginger is one of the herbal drugs which are effective for the treatment of nausea and vomiting without any special side effect. This herb is used in Pharmacoopeh, Germany to provide the anti-vomiting drugs (7).

The major pharmacological activity of ginger, Zingiber official, is related to its active components named of gingered and shogalos. The effects of these combinations are anti inflammation, anti vomiting, anti fever, anti cough, anti blood pressure, anti cancer, decreasing of prostaglandin, and the sedation of digestive problems. The effect of ginger products as an antiemetic is implemented by several mechanisms. For example, ginger and shogaols decrease the stomach contractions, but increase the activity of gastro intestinal trace (GIT). These combinations have anti-cancer effect and exert the effects of garbage against of the free radicals (8). Sontaki, et al. has done a research on using ginger on 50 cancer patients under chemotherapy. The subject of this study was, “Ginger, an antiemetic factor in nausea and vomiting induced by the chemotherapy”. They showed that ginger is more effective than Metoclopramid in controlling of nausea and vomiting. Their findings need more researches in order to prove ginger potential as an antiemetic drug for controlling vomiting induced by chemotherapy and recommend it as a cheaper solution (9). Abolghasemi et al. also did a research on 44 women with the first pregnancy who were afflicted by the nausea and vomiting. The aim of this study was determination of the ginger’s effect on nausea and vomiting during pregnancy. Their efforts showed that consuming 750 mg of ginger daily is an appropriate method to improve the nausea and vomiting during pregnancy (7).

There are some scientific studies in which contradictory findings have been reported. The effects of ginger on nausea and vomiting after surgery by laparoscopy genital system on 180 under surgery women indicates that the ginger had no effect on the intensity of nausea and vomiting in comparison with control group (10). Furthermore, Manusirraithaya et al. did a research on 43 cancer patients under chemotherapy with the aim of determining the effects of ginger’s animatic on chemotherapy nausea and vomiting. They showed that ginger is effective in decreasing of nausea and chemotherapy vomiting in delayed phase. It’s important to mention that in the current study there wasn’t any difference in acute phase of nausea and vomiting first 24 between groups treated by ginger and ones received placebo (8).

## 2. Objectives

With consideration to contradictory results of antiemetic effects in different studies, this study was aimed to determine the effect of ginger on chemotherapy induced nausea and vomiting in cancer patients.

## 3. Material and Methods

This study is a randomized, prospective, cross-over double - blinded clinical trial, conducted on patients under chemotherapy treatment. Subjects of this study were selected from cancer patients attending in hematology ward in university hospital. In this research information was gathered by using questions and check list after the following steps:

1)Supplying and editing the check list

2)Determining the validity and reliability

3)Presenting the letter of introduction from university.

4)Acquiring the permission from behavior committee. Hospital authority Oncology specialist, center of blood and patient research (acquiring the form of conscious satisfaction)

During the study the researcher attended in hematology ward during the week from Saturday to Thursday from 8 am. For about 6.5 month (from September 1st until March 20th 2007). After interviewing with available patients and filling up the information form, she was making certain that the chosen person has the appropriate conditions to be a sample for the research. This study was doing on the basis of the block randomization with the four block method. The researcher was choosing A and B regimes and evaluating the effects of these two regimes on the patients who were under chemotherapy on the basis of the selected group of the plan. The A regime was a routine antiemetic drugs along with 4 ginger capsules. Two of them should be taken 30 minutes before prescription of chemotherapy in the edible form. The other capsules were received 6 hours after chemotherapy.

The B regime was a routine antiemetic along with 4 placebo capsules. Two of them 30 minutes before the chemotherapy prescription and two others were received 6 hours after chemotherapy. Routine antiemetic regimen consists of one Ampoule of Grainestrone (keitril) 3 milligrams and one Ampoule of Degzametazone of 8 mg which patient was received 30 minutes before chemotherapy as basic drug for controlling chemotherapy nausea and vomiting. This drug may be used with 10 mg Metocolopramid which was prescribed within 24 hours, if it was requested by the patient.

It is necessary to explain that ginger capsule, with Zintoma trade mark, consists of 250 mg ginger powder which was produced by Isfahan Gol daru and placebo consists of 250 mg ineffective powder (Chickpea powder). The shape, color, and fragrance of this powder were similar to ginger and both of them were provided by the same company. Patients were chosen on the basis of inclusion criteria containing the following items:

1)Age over 18

2)Having the experience of chemotherapy with nausea and vomiting,

3)Having at least two chemotherapy episodes. Consists of 50-100 mg Cisplatin, with or without other chemotherapy agents with similar amount and prescription in two chemotherapy cycles without having any plans for radiotherapy among cycles

4)Presence in ward for 24 hours ( in order to investigate the PRN)

5)Having no nausea and vomiting experiences for some reasons except for chemotherapy,

6)Lack of receiving PRN in 24 past hours,

7)Lack of treatment with corticosteroid drugs during considered cycles,

8)Lack of afflictions to hepatitis, digestion system blockage, brain malignancy and cerebral metastasis and clotting disorder on the basis of recorded information in files

9)Lack of using the anti-clot drug.

The style of assigning scores to strain tool of intensity of vomiting and the way of investigation, by kortila tools was explained to the patient. If a patient was under radiotherapy or the process of investigation was stopped for any reason (nausea and intensive vomiting and the elimination of capsules and lack of re swallowing ingestion) the trend was, he or she left out of the plan. The process of patient's inspection was as follows. The researcher gave two edible 250mg capsules (ginger or placebo) to the patient half an hour before chemotherapy and simultaneously with Prescription Ampoule ketril 3 mg and Dexamethazon 8mg. afterwards gave two similar edible capsules 6 hours after chemotherapy. As mentioned, the capsules were chosen so that the patient received one kind of regimen during each cycle. Thus finally, all of the patients who were under investigation received both regimes A and B randomly, at least for 28 days, during two episodes.

The nausea's frequencies and score were measured 5 times during 24 hours. These measurements were done 1,2,3,4 hours after prescription of the second dose of capsules and 24 hours after chemotherapy in the next morning. In each time the severity of the measurement was determined by using the strain tools of severity of nausea. In this tool the flat surface or zero equals to the lack of nausea existence, steps 1 to 3 equal to mild nausea, steps 4 to 6 equal to average nausea, steps 7to 9 equals to sever nausea and step 10 equals to the severest possible nausea. The period of vomiting and retching was also observed during 24 hours, simultaneously with observation of nausea severity. The severity was also determined by Kortilla tools. The measurement of the severity of vomiting by using this tool was as follows:

•no retching equals to the lack of vomiting ,

•less than 3 times of retching with or without eliminating of stomach content equals to mild vomiting ,

•3 to 5 times of retching with or without the eliminating of stomach content equals to average vomiting

•More than 5 times of retching with or without eliminating of stomach content equals to sever vomiting

**Figure 1.**
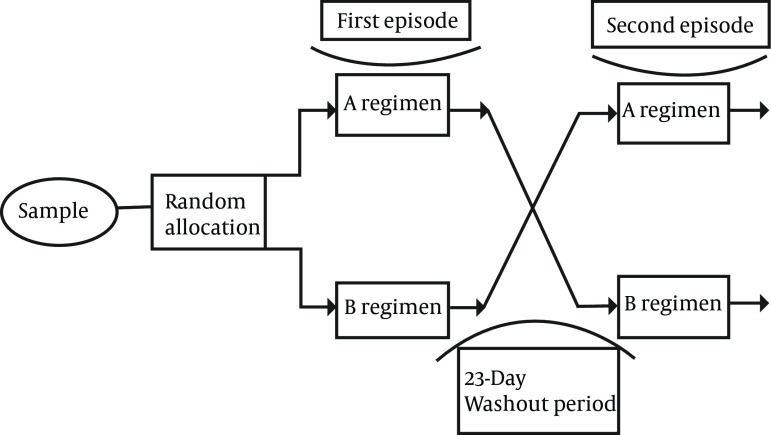
The Schematic Diagram of the Study Sequences

It is necessary to mention that within the hours of measurement of severity of the nausea and vomiting and at the end of 24 hours, Methocolopramid does which was used by the patient as PRN, was investigated and recorded in check list. In this research the effect of period, group and treatment was measured, in a way that the difference, sum of the average and the standard deviation of nausea score (the frequency of vomiting) the severity of nausea ( the intensity of vomiting ) was measured as (Table 1 ) in periods 1 and 2 (regimen A and B). Figure 1 shows the schematic diagram of study sequences.

**Table 1:** Treatment, Group and Episode Effects in Methods

Treatment Effect	Group Effect	Period Effect
**first episode A regimen – second episode B regimen**	(second episode B regimen + first episode A regimen) ÷ 2	first episode A regimen – second episode B regimen
**first episode B regimen - second episode A regimen**	(second episode A regimen + first episode B regimen) ÷ 2	two episode A regimen - first episode B regimen

Finally, the information was analyzed statistically by using SPSS version 16, in a way that the description was applied the data descriptive statistic descriptive and categorize and comprehensive statistic was used to test the hypothesis. Kolmogorov - Smirnov Test was applied to prove adaption to normal distribution. The independent t-test and data was used to analyze the effect of treatment, period and group in data with normal distribution and for data without normal distribution non-parametric test (Mann- Whitney U test) was implemented.

## 4. Results

The numbers of measured samples during the first cycle were 44, 22 received regime A and 22 received regime B. In the second cycle, 31 patient were measured by researcher and other could not complete the study because; Death of 3 people from samples, the change of drug regime (Two persons), vomiting and capsule elimination and lack of capsule swallowing (Two person), and relinquishment from continuing the treatment by a person without any special description. Five people of participants were under radiotherapy between the cycles of chemotherapy, which this situation was also considered as exclusion criteria, so these samples were left out of the study. Findings showed that most of the researched units (56.8%) were over 50 years (50.3 ± 13.1), (%59.1) male and many of units afflicted with the esophagus cancer and most of them (%47.7) were under therapy with 60 - 69 mg /m^2^ of Cisplatin. Most of the units in the first cycle (%81.8) and the second cycle (%46.6) had 2 - 4 times chemotherapy period and %52.3 received Cisplatin in combination with Fluorouracil 5. The intensity of acute phase nausea (24 hours) in different cycles for patient who received regime A (routine anti – emesis regime and ginger) and regime B (routine anti emesis regime and placebo) was observed. These measurements showed that the severity of the nausea in the first, second, third, fourth and the end of 24 hours in people who received regime A rather than regime B reduced %9, %18.2, %13.7, %22.7, %13.7, %27.3 respectively. In the second cycle these values were %7.2, %8 (in regime B) %5.3 , %14.1 (in regime B) and %24.1. About acute phases of vomiting, in the considered cycles, for patients who received regime A (Routine anti-emesis and ginger) and regime B (Routine anti-emesis and placebo) were studied. The results showed that the severity of the vomiting in the first, second, third, fourth hours and the end of 24 hours, in the first cycle of measurement for people who received ginger were respectively %9.1, %9.1, %9.1, %4.6,%4.7 less than (placebo). The results of the second cycle were showed that in the first hour there wasn’t any significant difference between the intensity of vomiting in two groups. In second, third, fourth hours and at the end of 24 hours respectively %4, %6.3, %6.7 and % 8.3 decreases have been seen in regime B in comparison the regime A. The frequencies of nausea in the first, second, third, fourth hours and at the end of 24 hours were measured. Then the measurement of the lack of group and period effect, and the effect of treatment were measured with statistical Mann- Whitney U test. There wasn’t any significant statistical difference between two groups in two cycles of treatments. Nausea scores in the first, second, third and fourth hours were measured. After elimination of the group and period effects, the effect of treatment was measured. In these hours the nausea score in ginger group also decreased, but there wasn’t any statistical significant difference between two groups in the mentioned hours. Nausea score in 3rd and from 4th hour to the end of 24 hours was measured. The effect of group and period was measured in the mentioned hours. After making certain of the lack of their effects, the effect of treatment was measured. These measurements became meaningful after using independent t test Mann – Whitney U test with P < 0.01 and P < 0.05 (Tables 2 and 3). The frequencies of vomiting were also measured in accordance with the findings of in the 1st, second, third, fourth hours and at the end of 24 hours. After observing no significant difference between group and period effects, the effect of the treatment was measured. In this measurement no significant difference was also appeared in all of the mentioned hours.

**Table 2:** Treatment,Group and Period Effect on the Nausea Score at the End of 24 Hours

	Nausea score at the end of 24 hours in both period	Mean ± SD	Results
**Treatment effect**	first episode A regimen – second episode B regimen	- 0.93±3.39	t^a^= -2.571, df = 28, p = 0.01
	first episode B regimen - second episode A regimen	1.80±2.33	
**Group effect**	(second episode B regimen+ first episode A regimen) ÷ 2	1.33 ±1.25	T = 0.07, df = 28, N/S
	(second episode A regimen + first episode B regimen)÷ 2	1.30±1.36	
**Period effect**	first episode A regimen – second episode B regimen	-0.93±3.39	T = 0.815, df = 28, N/S
	second episode A regimen - first episode B regimen	-1.80 ±2.33	

**Table 3:** Treatment, Group and Period Effect on the Nausea Score 3 HoursAfter Prescription

	Nausea score 3 hours after prescription in both period	Mean ± SD	Results
**Treatment effect**	first episode A regimen – second episode B regimen	-0.46 ±2.03	U^a^=77; df=28; P=0.05
	first episode B regimen - second episode A regimen	1.13±1.96	
**Group effect**	(second episode B regimen+ first episode A regimen)÷ 2	0.96±2.02	U=108; df=28; N/S
	(second episode A regimen + first episode B regimen)÷ 2	0.96±1.89	
**Period effect**	first episode A regimen – second episode B regimen	-0.46±2.03	U=93.50; df=28; N/S
	second episode A regimen - first episode B regimen	1.133±1.96	

## 5. Discussion

The findings of this research showed that the severity of the nausea in acute phase (the first 24 hours) in the first and second cycles in ginger consignee group was less that placebo. About the effectiveness of ginger in different hours of measured cycles can refer to the study, done by Nanthakoma et al. The results of this study showed that in nausea was appeared in %48.3 of ginger group members and %66.7 of placebo group members, so the ginger was 18.4% more effective than placebo (11).

Another approving study is the study which is done by Vutyavanich et al, The results of this study showed that , the decrease of nausea average score for all participants in ginger group was more than place. In this research after receiving the considered regime, the improvement of nausea was 28 out of 32 people in ginger group (%87.5)and 10 out of 35 people in placebo (%28.2) (12).With regard to the severity of vomiting of sever phase of the measured cycles in patients received regime A (routine and antiemetic regimen plus ginger) and regimen B (routine antiemetic plus placebo), the results show that the intensity of vomiting in the first, second, third, fourth hours, and at the end of 24 hours of the first measuring cycle in people received ginger was respectively %9.1, %9.1,%9.1, %4.6 and %4.7 less than placebo. In this case we can refer to the research was done by Vutyawanich et al., in which the reduction of Vomiting period in ginger consignee with the effectiveness of %28, 2 was appeared (12).

The result in second cycle showed that in the first hour there wasn’t any difference between the intensity of vomiting in two regimes. There was respectively %4, %6.3, %6.7,%8.3 reduction in regimen A compared to regimen B for second , third , fourth hours and at the end of 24 hours. The study done by Visalyputra was from researches in which the effect of placebo was considerable. In this study there was no difference in the amount of nausea and vomiting incidence between placebo and ginger users. The results of the mentioned study showed that nausea incidence for placebo was %32, for ginger users was %22, and for dipridamol and ginger users was %33. For vomiting these values were %32, %25 and %25 respectively. Thus the amount of nausea and vomiting incidence in placebo and ginger group were 2 gram and for dipridamol was similar to each other and there wasn’t any difference (13).

The of the frequencies of nausea in the first, second, third, fourth hours and at the end of 24 hours, after omitting of the effect of group and period, by using Mann - Whitney U test in measuring the effect of therapy presented the lack of significant statistical difference. These results were similar to the results gained by Leopold et al. In the mentioned study the nausea incidence after operation were %49, 58%, and 53% respectively for placebo group, 300 and 600 mg ginger group, in which ginger group the effectiveness of therapies wasn’t significant P = 0.69 (10). In this study similar to the frequencies of nausea, there was no significant difference in score of nausea. This result was similar to Apariman et al. aim of findings. Apariman et al. showed that in the second hour after the surgery operation the average nausea score in ginger group was zero and in placebo was 0.15 that at this moment the change of nausea score in two groups wasn’t significant with P = 0.142 (14).

Nausea score in the third hour and from the fourth hour to the end of 24 hours was also measured. In the mentioned hours and after measuring the effect of period and group and after certainty of the lack of their effect, the effect of therapy measured. By using the Mann - Whitney U test were respectively significant with P < 0.01 and P < 0.05. Here we can refer to the research done by Sontaki et al. This research presents the reduction of nausea score for %62 of under therapy with ginger that was significant from the statistical point of view (9). The frequencies of vomiting on the basis of findings in the first, second, third, fourth hours and at the end of 24 hours were measured in both cycles. After measuring the effect of group and period, that didn’t show significant difference, the effect of therapy was measured. In none of the hours of measuring significant statistical difference was appeared, the results of this study were similar to Visalyapotra research’s results. The patients were categorized in four groups including placebo, placebo with Deropridol drug, ginger, and ginger with Deropridol drug. Afterwards the severity of their nausea and vomiting was measured two times in 24 hours. The results showed that there were no significant statistical difference for incidence of nausea and vomiting in the measured groups (13). In the research of Sontaki et al., the number of vomiting periods in the second 12 hours had significant increase in comparison with the first 12 hours. The researcher realized that its reason was the reduction of anti- vomiting effects of ginger after the passage of time (9). Moreover, the study done by loin et al. showed that ginger in conjunction with protein in the meal leads to the reduction of delay nausea and the reduction of using antiemetic drugs (15).

On the other hand, Pangrojpaw et al. showed that ginger was more effective than Diphenhydramin, in addition it has less side effects (16). Ozgoli et al. also emphasized on using ginger as an herbal drug for nausea and vomiting (17)

Conclusion: although there wasn’t any significant difference in the present research in all statistical tests between ginger and placebo, in most hours of measurement the reduction of intensity score and the frequencies of nausea were observed. This change was less in the frequencies of vomiting. Therefore, on the basis of present research and the other studies, done in all over the world, ginger can be used as a simple method, inexpensive and secure and as complimentary of antiemetic in controlling of nausea and vomiting. In this study, the lack of attainment to expected results can be assigned to factors such as the lack of sufficient ginger does’ sufficiency, the little amount of available sample, the lack of exclusiveness of the cancer, and the hours of giving ginger. Thus by implementation of widespread studies and with solving the mentioned problems, it is possible to gain the better results.
